# Metabolic Resistance in Abamectin-Resistant *Bemisia tabaci* Mediterranean from Northern China

**DOI:** 10.3390/toxins14070424

**Published:** 2022-06-22

**Authors:** Ran Wang, Yong Fang, Wunan Che, Qinghe Zhang, Jinda Wang, Chen Luo

**Affiliations:** 1 Institute of Plant Protection, Beijing Academy of Agriculture and Forestry Sciences, Beijing 100097, China; 202072776@yangtzeu.edu.cn (Q.Z.); luochen@ipepbaafs.cn (C.L.); 2 Agriculture Biotechnology Institute, Hunan Academy of Agricultural Sciences, Changsha 410125, China; yongfang@hunaas.cn; 3 Department of Pesticide Sciences, Shenyang Agricultural University, Shenyang 110866, China; chewunan@syau.edu.cn; 4 National Engineering Research Center of Sugarcane, Fujian Agricultural and Forestry University, Fuzhou 350002, China

**Keywords:** *Bemisia tabaci*, abamectin, cross-resistance, synergistic effects, metabolic enzymes, inheritance, resistance management

## Abstract

Abamectin, produced by the soil-dwelling actinomycete *Streptomyces avermitilis*, belongs to the macrocyclic lactones class of pesticides, has nematocidal, acaricidal, and insecticidal activity, and is highly effective when used against targeted species. *Bemisia tabaci*, the tobacco whitefly, is a highly destructive insect to agricultural production worldwide, and various insecticide-resistant strains have been identified in China. Here, we monitored levels of resistance to abamectin in twelve field-collected *B. tabaci* populations from northern China, and confirmed that, compared with the lab reference strain, six field populations exhibited strong abamectin resistance, while the other six exhibited low-to-medium resistance. Among these, the Xinzheng (XZ) population displayed about a 40-fold increased resistance to abamectin, and experienced significant cross-resistance to chlorpyrifos and imidacloprid. The abamectin resistance of XZ was found to be autosomal and incompletely dominant. Metabolic enzyme and synergism tests were conducted, and two metabolic enzymes, glutathione S-transferase and P450 monooxygenase, were found to be conducive to the field-developed abamectin resistance of the XZ population. The above results provide valuable information that can be used in identifying new pest control strategies and delaying the evolution of resistance to abamectin in field populations of whiteflies.

## 1. Introduction

Avermectins are natural products generated from fermentation by *Streptomyces avermitilis*. *S. avermitilis* is a soil bacterium, and the microbe-produced insecticidal toxins display excellent effects against various pests [1]. Due to being less toxic in the environment and having powerful activity for controlling mites and insects, among all the members of avermectins, it is well known that abamectin is one of the most extensively applied toxins against a series of insect pests around the world [2]. However, as with many other popular chemical agents, field-evolved resistance to abamectin has been monitored and recorded in a variety of insect pests worldwide [2], and elevated metabolic detoxification has been considered as one key mechanism of abamectin resistance in various mites and insects [3,4,5,6]. In one lab-selected abamectin-resistant population of the moth, *Plutella xylostella*, which exhibits a 23,670-fold resistance ratio, piperonyl butoxide (PBO) was found to inhibit abamectin resistance to a small extent, and P450 monooxygenase (P450s) activity was significantly elevated [4]. Similarly, in the thrips, *Frankliniella occidentalis*, assessments of synergism and assays of detoxifying enzymes indicated that enhanced activity of cytochrome P450s was the primary factor resulting in 45.5-fold resistance to abamectin after merely 15 times of selection [7]. Glutathione S-transferases (GSTs) were also found to exert crucial influence in promoting the detoxification against natural and synthetic xenobiotics for pest control and are related to the evolution of resistance to chemical agents in insect pests [8]. Metabolic assays indicated that the activity of GST in two abamectin-resistant populations of the vegetable leafminer, *Liriomyza sativae*, was greatly increased compared with the susceptible population, suggesting that enhanced activity of GST could be one primary factor conferring resistance to abamectin in this pest insect [9], and recently it was found that in *Chilo suppressalis*, the rice borer, inhibited activity of GST markedly elevated the susceptibility to abamectin [10].

*Bemisia tabaci*, the highly invasive and genetically diverse whitefly, is one notorious sucking insect globally, and it brings harm to not less than 600 species of host plants, both directly by feeding with their piercing–sucking mouthparts and indirectly through the transmission of various plant viruses [11,12]. Presently, the Mediterranean (MED or Q biotype) and Middle East–Asia Minor 1 (MEAM1 or B biotype) strains are confirmed as the most extensively distributed and invasive cryptic species [12]. In China, at the early stage, MEAM1 of *B. tabaci* was the main cryptic species known to cause heavy damage to a variety of crops with high economic value, but in 2003, infestation by the MED cryptic species was first found in horticultural crops [13,14,15], and currently, it has been demonstrated that MED has been considered as the dominant cryptic species instead of MEAM1 [16,17].

Although there are several other candidates for controlling *Bemisia tabaci*, like using predators *Amblyseius swirskii* and *Nesidiocoris tenuis*, the management of whiteflies rests primarily upon the use of chemical agents, and over time, many populations of *B. tabaci* from the field have evolved moderate to very-high resistance to popular chemical agents, making management of this pest increasingly challenging [15,18,19]. Specifically, approximately 650 reports of resistance to more than 60 insecticidal agents have been recorded in whiteflies [20], especially in the last five years, during which resistance to various commonly used insecticides such as cycloxaprid, cyantraniliprole, flupyradifurone, and spirotetramat has been recorded in different parts of China [21,22,23,24,25]. Therefore, widely and heavily applied insecticides can no longer be considered an effective, or even appropriate, means of controlling *B. tabaci* in China. As recorded formerly in a lot of other insect pests, field-selection pressure applied by long-term and continual applications of insecticides is likely conducive to the evolution of resistance in whiteflies. In the present work, we monitored resistance to abamectin from multiple populations of whiteflies collected throughout northern China and found the Xinzheng (XZ) field population exhibited moderate resistance to abamectin and significant cross-resistance to chlorpyrifos and imidacloprid. To explore the mechanism of resistance to abamectin, assays of synergism and metabolic enzyme were conducted in the XZ population, and tests of inheritance were also performed to characterize resistance to abamectin in the XZ population.

## 2. Results

### 2.1. Monitoring and Cross-Resistance Tests

The baseline of resistance to abamectin was measured in twelve populations collected from different regional parts of China in the year of 2021 (Table S1). In comparison with the reference strain MED-S, six of the twelve field-populations showed high susceptibility to abamectin with a resistance ratio that ranged from 1.0 to 4.6 fold (LC_50_: from 0.080 to 0.355 mg L^−1^). Another six populations showed low to medium resistance to abamectin, and among them, the Xinzheng (XZ) population displayed 42.6-fold resistance (LC_50_: 3.283 mg L^−1^) (Table 1), significant cross-resistance to chlorpyrifos (5.3-fold) and imidacloprid (5.9 fold), and no cross-resistance to bifenthrin (2.7 fold), flupyradifurone (1.3 fold), sulfoxaflor (1.5 fold), and thiamethoxam (2.0 fold) (Table 2).

### 2.2. Synergism Assays

The effects of synergism with PBO, DEM, and TPP in abamectin on the MED-S and XZ populations are presented in Table 3. Significant effects of synergism with DEM and PBO with resistance to abamectin were observed in XZ population, having synergistic ratios (SR) of 2.22 and 2.81, respectively; TPP exhibited little significant effects of synergism in the XZ population (SR = 0.95). The above data suggest that GSTs and P450s are likely associated with resistance to abamectin in the XZ population.

### 2.3. Metabolic Enzyme Activities

To assess further roles of detoxifying mechanisms in abamectin resistance, the activities of EST, P450, and GST were determined in the two tested populations (Table 4). Activity of P450 in the XZ population (elevated 2.25 fold) was significantly increased compared to MED-S; activity of GST in the XZ (elevated 3.09 fold) was also significantly increased compared to MED-S. Conversely, the activity of esterase towards α-naphthyl acetate was not greatly different between the MED-S and XZ population.

### 2.4. Inheritance of Abamectin Resistance in XZ Population

Results of dose–response in XZ, MED-S, and the F_1_ progenies are described in Table 5. For the XZ population with moderate abamectin resistance, little significant difference in the values of LC_50_ were observed between the F_1A_ progeny (LC_50_: 2.987 mg L^−1^), F_1B_ offspring (LC_50_: 2.621 mg L^−1^), and F_1_ pooled offspring (LC_50_: 2.929 mg L^−1^), implying that the abamectin resistance in XZ is inherited autosomally. Additionally, the dominance degree of F_1A_, F_1B_, and F_1_ pooled was, respectively, 0.84, 0.78, and 0.83, indicating that the abamectin resistance inheritance in the XZ population was incompletely dominant.

## 3. Discussion

Over the past ten years, abamectin has been considered a significantly efficacious chemical agents for controlling populations of whiteflies with high susceptibility, and it is presently available against field populations according to several previous reports [15,18,26]. In the current work, we collected twelve field populations of *B. tabaci* from different regional parts of northern China and detected that half of these field-collected populations still exhibited susceptibility to abamectin. However, another six field-collected populations showed low to medium resistance to abamectin, among which the (Xinzheng) XZ population displayed over 40-fold elevated resistance (LC_50_: 3.283 mg L^−1^) compared with the reference population MED-S. This is similar to what has been found in other studies of field-evolved resistance in China, in which abamectin resistance varied from 8- to 35.5-fold in field populations in comparison with the reference population [6]. Low to high abamectin resistance was also reported in various of field-collected populations of *Frankliniella occidentalis* and *Tetranychus urticae*, and in particular, field-collected populations of *T. urticae* displayed moderate to very high abamectin resistance, having a 316.67- to 1809.51-fold elevated resistance ratio, which suggests that a growing number of insect pests possibly developed insecticide resistance [27,28].

Significant effects of synergism in abamectin resistance with DEM and/or PBO, and increased activities of GSTs and/or P450s were observed in resistant populations of various insect pests [4,7,29,30]. Previously, it has been found that DEM, glutathione S-transferase inhibitor, and PBO, the oxidase inhibitor, caused remarkable synergisms with resistance in the abamectin-selected NJ-Abm population of *B. tabaci*, and elevated detoxification resulting from GSTs and P450s was also found to be associated with mediating resistance to abamectin in this strain [31]. Here, the effects of synergism mediated by inhibitors of metabolic enzymes on the toxicity of abamectin were significant in the XZ population of *B. tabaci*, indicating that metabolic detoxification could be the primary mechanism underlying the observed resistance. The present work also shows that enhanced metabolism mediated by P450s and GSTs could also be conducive to the evolution of resistance to abamectin in the field-collected XZ population. Therefore, in vivo assessments of synergism and in vitro assays of detoxifying enzymes indicated that enhanced activities of P450s and GSTs were the primary factors contributing to the development of resistance to abamectin. Additionally, considering that field-developed resistance to abamectin in various insect pests show diverse mechanisms, increased detoxifying metabolism may not always be the dominant mechanism of abamectin resistance, and target-site resistance could not be excluded [2,3,4].

It has been well demonstrated that cross-resistance patterns in insect pests could imply useful information in the management of resistance to popular chemical agents, and those screened with abamectin typically cause significant cross-resistance to other analogues of abamectin rather than other types of insecticide [2,31,32]. However, in our current study, significant cross-resistance to chlorpyrifos and imidacloprid was observed in the field-evolved, abamectin-resistant population. Similarly, moderate levels of cross-resistance to chlorpyrifos was observed in one abamectin-resistant population of *F. occidentalis*, while one lab-selected population of *B. tabaci* with a high level of abamectin resistance showed low cross-resistance to imidacloprid [7,31]. Owing to the potential threats from cross-resistance to chlorpyrifos and imidacloprid, these three chemicals are suggested to be considered as one individual group while implementing a rotation plan for using chemical agents in whiteflies management. Moreover, the above results on patterns of cross-resistance could be helpful for setting and implementing the rotation plan to postpone the development of resistance to abamectin in field-collected populations of whiteflies.

Our current work found that abamectin resistance-related genes of *B. tabaci* belong to autosomal chromosomes and abamectin resistance in *B. tabaci* is evaluated as one dominant trait. Imidacloprid, flupyradifurone, and cyantraniliprole resistance in whiteflies were also found to be autosomally inherited [33,34,35]. Moreover, the dominance degree could be varied depending on the type of chemical agents, genetic backgrounds, species of insect, state of the environment, and records of historical selections [36]. For instance, imidacloprid and cyantraniliprole resistance in *B. tabaci* displayed incompletely dominant traits [33,34], whereas flupyradifurone resistance in whiteflies is incompletely recessive [35]. While the concentration used is not enough for killing heterozygous specimens, the codominant trait of resistance to chemical agents probably become the functionally dominant trait, and it is important to enable the maintenance of susceptible genes [36,37]. Under these circumstances, the functionally dominant trait of resistance to abamectin in *B. tabaci* probably causes the resistance to be unsteady in the field. Resistance to chemical agents governed by functionally dominant or dominant alleles may evolve sooner than resistance governed by recessive genes, as homozygous and heterozygotes insects are less likely to die when exposed to chemical agents in the field [37]. The inheritance of abamectin resistance detected in our research can be utilized when formulating strategies for insecticide resistance management. Once abamectin resistance evolves in the field, the application is suggested to be suspended instantly and replaced by insecticides that show little cross-resistance. Subsequently, abamectin application could be suggested while activities of monitoring resistance uncover that the susceptibility of *B. tabaci* to abamectin has been reverted.

## 4. Materials and Methods

### 4.1. Insects

The reference population of *B. tabaci* MED was initially sampled from poinsettia *Euphorbia pulcherrima* in Beijing and had not been exposed to insecticides in over ten years [16]. The twelve field populations of whiteflies were sampled from across different regional parts of northern China (Table S1), and all of them were determined to be the MED cryptic species based on one reported step [13]. All populations from the field were fed on plants of cotton *Gossypium hirsutum*, and maintained on the cotton plants in individual rearing rooms until assays with the temperature setting at 26 ± 2 °C, the relative humidity setting at 55 ± 5%, and the photoperiod set as 14:10 h light and dark. For bioassays of the tested chemical agents, adults of *B. tabaci*, aged up to seven days post eclosion, were collected randomly.

### 4.2. Insecticides and Chemicals

The following commercial insecticides were utilized in the bioassays: abamectin, 18 g L^−1^ emulsifiable concentrate (Agrimec, Hebei Veyong Biochemical Co., Ltd., Shijiazhuang, China); bifenthrin, 100 g L^−1^ emulsifiable concentrate (Capture, Bayer Cropscience China Co., Ltd., Beijing, China); chlorpyrifos, 480 g L^−1^ emulsifiable concentrate (Lorsban, Dow AgroScience, Beijing, China); flupyradifurone, 17% soluble concentrate (Sivanto, Bayer Cropscience China Co., Ltd., Beijing, China); imidacloprid, 100 g L^−1^ suspension concentrate (Gaucho, Bayer Cropscience China Co., Ltd., Beijing, China), sulfoxaflor, 22% suspension concentrate (Transform, Dow AgroScience, Beijing, China); and thiamethoxam, 250 g L^−1^ water dispersible granule (Actara, Syngenta Crop Protection Company, Shanghai, China). Triphenyl phosphate (TPP), diethyl maleate (DEM), and piperonyl butoxide (PBO), dimethyl sulfoxide (DMSO), and Triton X-100 were bought from Sigma Aldrich, Shanghai, China.

### 4.3. Bioassays and Tests of Synergism

All of the whitefly bioassays were performed according to the described steps [23]. Specifically speaking, all of the chosen commercialized insecticides were dissolved and stock solutions (1000 mg/L) of all tested insecticides were prepared. Then, different concentrations of the working solutions were prepared by diluting the stock solution in 0.1% Triton X-100 in distilled water, and five tested concentrations of each chemical agent were used in the bioassays. Next, 20 mm diameter cotton leaf discs were dipped in each replicate of the tested concentration of each insecticide for twenty seconds, and four replicates were set up for each tested concentration. Control leaf discs were dipped in 0.1% Triton X-100 as described above, and four replicates were made for each concentration. The cotton leaf discs for the bioassays were dried at room temperature, and after that were put onto 1.8 mL of agar (15g L^−1^) in one 60 mm-long test tube. In each one of the test tubes, 25–30 adult whiteflies were introduced, and then kept in the incubators with the temperature setting as 26 ± 2 °C the relative humidity setting as 55 ± 5%, and the photoperiod set as 14:10 h light and dark. In each one of the bioassays, adult whitefly mortality was checked after 48 h using a microscope, with motionless whiteflies being considered as dead ones. The data of bioassays, such as the LC_50_ values of the tested chemical agents, their 95% fiducial limits, and slopes ± SE were analyzed via probit analysis, and mortality data for *B. tabaci* were corrected using Abbott’s formula using the software of PoloPlus (LeOra Software, Berkeley, CA, USA, 2002). The resistance ratio (RR) of each chemical agent against *B. tabaci* was calculated by dividing the LC_50_ value of the field population by the LC_50_ value of the reference strain, and the RR values were utilized to present levels of resistance [26].

### 4.4. Metabolic Enzyme Assays

The activities of glutathione S-transferases (GSTs), esterases (ESTs), and P450 monooxygenases (P450s) were measured on the basis of one reported approach with little change [25]. About 500 adult whiteflies were collected at random and homogenized in 0.5 mL phosphate buffer (0.1 M, pH 7.8) containing 1 mM PMSF, 1 mM PTU, 1 mM DTT, and 1 mM EDTA at 4 °C. The homogenate was centrifuged for 15 min at 11,000× *g* at 4 °C. The supernatant was sampled and further centrifuged for 30 min at 16,000× *g* at 4 °C. The supernatant was again sampled and utilized as an enzyme source for detecting the activity of P450s. For measuring activities of ESTs and GSTs, about 400 adult whiteflies were collected at random and homogenized in 0.5 mL phosphate buffer (0.1 M, pH 7.5). The homogenate was centrifuged at 11,000× *g* for 10 min at 4 °C, after which the supernatant was sampled and utilized as an enzyme solution, and the content of total protein for all enzyme solutions was measured based on Bradford’s method [38].

### 4.5. Inheritance Tests

To determine inheritance of the insecticide-resistant phenotype of the XZ population, each of the tested straina was crossed reciprocally with the reference population MED-S according to the reported steps [33]. For each tested strains, approximately 120 pseudopupae were sampled randomly and individually put into the 96-well microplate and sealed by parafilm. Then, freshly emerged males and females from each strain were collected from 24 to 48 h. For the reciprocal crosses, tested adults were kept on cotton leaves in one clip cage and allowed to lay eggs for 6-days. The offspring produced by the MED-S ♂ × XZ ♀ and XZ ♂ × MED-S ♀ crosses were respectively termed F_1A_ and F_1B_. According to one published method [39], levels of dominance were calculated, and dominance degree (*D*) was evaluated for each test, respectively, based on the LC_50_ of XZ and MED-S, and their F_1_ progenies.

## Figures and Tables

**Table 1:** Abamectin resistance in field-collected *B. tabaci* populations from China.

Population	N ^a^	Slope ± SE	LC_50_ (95% CL) (mg L^−1^) ^b^	X^2^ (df)	RR ^c^
MED-S	547	1.038 ± 0.136	0.077 (0.057–0.098)	2.270 (3)	
LY	552	1.098 ± 0.135	0.159 (0.126–0.210)	1.355 (3)	2.1
CY	570	1.503 ± 0.141	0.080 (0.067–0.095)	1.972 (3)	1.0
HD	563	1.671 ± 0.146	0.093 (0.079–0.109)	1.879 (3)	1.2
TZ	566	1.553 ± 0.150	0.569 (0.455–0.683)	0.552 (3)	7.4
WQ	559	1.394 ± 0.145	0.614 (0.481–0.748)	0.858 (3)	8.0
JH	549	1.618 ± 0.157	1.088 (0.868–1.306)	1.387 (3)	14.1
ZJK	531	1.741 ± 0.159	0.355 (0.295–0.417)	1.930 (3)	4.6
BD	535	1.289 ± 0.149	0.460 (0.331–0.587)	2.018 (3)	6.0
ZZ	548	1.562 ± 0.168	0.147 (0.108–0.184)	2.692 (3)	1.9
XZ	561	1.236 ± 0.140	3.283 (2.424–4.142)	2.269 (3)	42.6
JN	557	1.215 ± 0.148	0.275 (0.181–0.368)	1.910 (3)	3.6
TA	554	1.445 ± 0.149	0.441 (0.342–0.539)	1.820 (3)	5.7

^a^ Number of insects used. ^b^ CL = confidence limits. ^c^ RR (resistance ratio) = LC_50_ (field-collected population)/LC_50_ (MED-S).

**Table 2:** Resistance spectrum of the susceptible (MED-S) and abamectin resistant (XZ) strains of *B. tabaci*.

Insecticide	Strain	N ^a^	LC_50_ (mg L^−1^) (95% CL) ^b^	Slope ± SE	X^2^ (df)	RR ^c^
Abamectin	MED-S	555	0.089 (0.069–0.110)	1.183 ± 0.137	2.052 (3)	
	XZ	565	3.472 (2.701–4.316)	1.152 ± 0.133	1.418 (3)	39.0
Bifenthrin	MED-S	552	124.472 (102.999–152.889)	1.350 ± 0.140	2.396 (3)	
	XZ	567	330.187 (271.711–392.142)	1.528 ± 0.143	1.556 (3)	2.7
Chlorpyrifos	MED-S	573	182.271 (142.653–221.115)	1.637 ± 0.159	1.287 (3)	
	XZ	559	963.926 (765.662–1187.025)	1.208 ± 0.136	1.866 (3)	5.3
Flupyradifurone	MED-S	574	15.490 (12.413–18.747)	1.348 ± 0.138	1.582 (3)	
	XZ	559	31.396 (26.161–36.933)	1.645 ± 0.150	2.207 (3)	1.3
Imidacloprid	MED-S	567	15.188 (12.897–17.590)	1.866 ± 0.156	1.951 (3)	
	XZ	571	89.592 (77.006–102.825)	2.010 ± 0.160	2.003 (3)	5.9
Sulfoxaflor	MED-S	568	11.540 (8.970–14.215)	1.251 ± 0.138	1.191 (3)	
	XZ	562	17.866 (15.185–20.775)	1.752 ± 0.151	1.786 (3)	1.5
Thiamethoxam	MED-S	564	10.310 (8.504–12.356)	1.404 ± 0.139	1.846 (3)	
	XZ	575	21.124 (17.380–24.956)	1.651 ± 0.151	2.353 (3)	2.0

^a^ Number of insects used. ^b^ CL = confidence limits. ^c^ RR (resistance ratio) = LC_50_ (XZ)/LC_50_ (MED-S).

**Table 3:** Synergistic effects of abamectin toxicity on the susceptible (MED-S) and abamectin-resistant (XZ) strains of *B. tabaci*.

Strain	Insecticide/Synergist	LC_50_ (mg L^−1^) (95% CL) ^a^	Slope ± SE	X^2^ (df)	SR ^b^
MED-S	Abamectin	0.094 (0.076–0.113)	1.377 ± 0.141	2.896 (3)	
	Abamectin + PBO	0.082 (0.067–0.099)	1.408 ± 0.114	2.452 (3)	1.15
	Abamectin + DEM	0.116 (0.094–0.139)	1.627 ± 0.152	2.198 (3)	0.81
	Abamectin + TPP	0.097 (0.070–0.124)	1.227 ± 0.145	1.583 (3)	0.97
XZ	Abamectin	3.887 (3.171–4.683)	1.391 ± 0.139	1.397 (3)	
	Abamectin + PBO	1.754 (1.255–2.243)	1.274 ± 0.147	2.349 (3)	2.22
	Abamectin + DEM	1.381 (1.097–1.688)	1.280 ± 0.138	1.257 (3)	2.81
	Abamectin + TPP	4.095 (3.348–4.947)	1.341 ± 0.139	1.492 (3)	0.95

^a^ CL = confidence limits. ^b^ SR (synergistic ratio) = LC_50_ (abamectin only)/LC_50_ (abamectin + synergist).

**Table 4:** Metabolic enzyme activities in MED-S and XZ *B. tabaci* populations ^a^.

Population	P450s Activity pmol min^−1^ mg^−1^	Ratio ^b^	ESTs Activity nmol min^−1^ mg^−1^	Ratio ^b^	GSTs Activity nmol min^−1^ mg^−1^	Ratio ^b^
MED-S	0.75 ± 0.14 a		296.55 ± 23.81 a		44.92 ± 9.76 a	
XZ	1.69 ± 0.18 b	2.25	312.14 ± 27.25 a	1.05	138.62 ± 15.38 b	3.09

^a^ Mean activity values in the same column followed by different letters are significantly different *(p <* 0.05). ^b^ Ratio = XZ activity/MED-S activity.

**Table 5:** Efficacy of abamectin treatment in susceptible (MED-S) and resistant (XZ) strains of *B. tabaci* and their F_1_ progeny from reciprocal crosses.

Population or Cross	LC_50_ (mg L^−1^) (95% CL) ^a^	Slope ± SE	X^2^ (df)	RR ^b^	*D* ^c^
MED-S	0.085 (0.068–0.103)	1.330 ± 0.139	1.069 (3)	1	
XZ	4.030 (3.520–4.585)	2.138 ± 0.163	1.528 (3)	47.4	
F_1A_ (MED-S ♂ × BD ♀)	2.987 (2.260–3.706)	1.369 ± 0.148	2.735 (3)	35.1	0.84
F_1B_ (BD ♂ × MED-S ♀)	2.621 (2.063–3.191)	1.406 ± 0.145	2.044 (3)	30.8	0.78
F_1_ (pooled)	2.929 (2.391–3.587)	1.264 ± 0.136	2.426 (3)	34.5	0.83

^a^ CL = confidence limits. ^b^ RR (resistance ratio) = LC_50_ (XZ or F_1_)/LC_50_ (MED-S). ^c^ The degree of dominance (*D*) ranges from −1 (completely recessive) to +1 (completely dominant).

## Data Availability

Most of the recorded data are available in the Tables in the manuscript.

## Supplementary Materials

The following supporting information can be downloaded at: https://www.mdpi.com/article/10.3390/toxins14070424/s1. Table S1: Information of field-collected *Bemisia tabaci* samples from northern China.
