# Supplementary material for: Metabolic Resistance in Abamectin-Resistant Bemisia tabaci Mediterranean from Northern China
Source: Toxins (Basel). 2022 Jun 22;14(7):424. doi: 10.3390/toxins14070424 (PMC9317228; doi:10.3390/toxins14070424)
Supplement: Supplementary file 1 [file toxins-14-00424-s001.zip › toxins-1769218-supplementary.pdf]

toxins

Metabolic Resistance in Abamectin-Resistant *Bemisia tabaci* Mediterranean from Northern China

Ran Wang, Yong Fang, Wunan Che, Qinghe Zhang, Jinda Wang and Chen Luo

Table S1. Information of field-collected *Bemisia tabaci* samples from northern China.

Names of populations	Locations of collection	Sites of collection	Date	Host plant
LY	Liaoyang, Liaoning	41.19N, 123.11E	Aug 2021	Eggplant
CY	Chaoyang, Liaoning	41.59N, 120.50E	Aug 2021	Cucumber
HD	Haidian, Beijing	39.97N, 116.31E	Apr 2021	Tomato
TZ	Tongzhou, Beijing	39.73N, 116.69E	Jun 2021	Tomato
WQ	Wuqing, Tianjin	39.35N, 117.10E	Jun 2021	Tomato
JH	Jinghai, Tianjin	38.90N, 116.94E	Jun 2021	Tomato
ZJK	Zhangjiakou, Hebei	40.58N, 115.00E	July 2021	Pepper
BD	Baoding, Hebei	38.82N, 115.39E	July 2021	Tomato
ZZ	Zhengzhou, Henan	34.91N, 113.56E	July 2021	Cucumber
XZ	Xinzheng, Henan	34.33N, 113.75E	July 2021	Pepper
JN	Jinan, Shandong	36.78N, 117.23E	Aug 2021	Tomato
TA	Taian, Shandong	36.14N, 117.22E	Aug 2021	Tomato
